# The Characterization of Immunoprotection Induced by a cDNA Clone Derived from the Attenuated Taiwan Porcine Epidemic Diarrhea Virus Pintung 52 Strain

**DOI:** 10.3390/v10100543

**Published:** 2018-10-04

**Authors:** Chi-Fei Kao, Hue-Ying Chiou, Yen-Chen Chang, Cheng-Shun Hsueh, Chian-Ren Jeng, Pei-Shiue Tsai, Ivan-Chen Cheng, Victor Fei Pang, Hui-Wen Chang

**Affiliations:** 1 School of Veterinary Medicine, National Taiwan University, Taipei 10617, Taiwan; fei81005@gmail.com (C.-F.K.); chyenjean@hotmail.com (Y.-C.C.); crjeng@ntu.edu.tw (C.-R.J.); psjasontsai@ntu.edu.tw (P.-S.T.); ivancheng@ntu.edu.tw (I.-C.C.); pang@ntu.edu.tw (V.F.P.); 2 Graduate Institute of Veterinary Pathobiology, College of Veterinary Medicine, National Chung Hsing University, 250 Kuo Kuang Rd, Taichung 402, Taiwan; hic01.chiou@gmail.com; 3 Graduate Institute of Molecular and Comparative Pathobiology, School of Veterinary Medicine, National Taiwan University, Taipei 10617, Taiwan; sam903124@gmail.com

**Keywords:** PEDV, infectious clone, reverse genetics, attenuation, immunoprotection

## Abstract

The porcine epidemic diarrhea virus (PEDV) poses a great threat to the global swine industries and the unreliable protection induced by the currently available vaccines remains a major challenge. We previously generated a genogroup 2b (G2b) PEDV Taiwan Pintung 52 (PEDVPT) strain, PEDVPT-P96, and determined its promising host immune response against the virulent PEDVPT-P5 strain. To study the attenuation determinants of PEDVPT-P96 and establish a PEDVPT-P96-based recombinant vector as a vaccine platform for further antigenicity modification, iPEDVPT-P96, a full-length cDNA clone of PEDVPT-P96, was established. Comparing to the parental PEDVPT-P96 virus, the iPEDVPT-P96 virus showed efficient replication kinetics with a delayed decline of viral load and similar but much more uniform plaque sizes in Vero cells. In the 5-week-old piglet model, fecal viral shedding was observed in the PEDVPT-P96-inoculated piglets, whereas those inoculated with iPEDVPT-P96 showed neither detectable fecal viral shedding nor PEDV-associated clinical signs. Moreover, inoculation with iPEDVPT-P96 elicited comparable levels of anti-PEDV specific plasma IgG and fecal/salivary IgA, neutralizing antibody titers, and similar but less effective immunoprotection against the virulent PEDVPT-P5 challenge compared to the parental PEDVPT-P96. In the present study, an infectious cDNA clone of an attenuated G2b PEDV strain was successfully generated for the first time, and the in vitro and in vivo data indicate that iPEDVPT-P96 is further attenuated but remains immunogenic compared to its parental PEDVPT-P96 viral stock. The successful development of the iPEDVPT-P96 cDNA clone could allow for the manipulation of the viral genome to study viral pathogenesis and facilitate the rapid development of effective vaccines.

## 1. Introduction

The porcine epidemic diarrhea virus (PEDV) is an enveloped, positive-sense and single-stranded RNA virus, belonging to the order *Nidovirales*, family *Coronaviridae*, and genus *Alphacoronavirus* [1]. The genome of PEDV is about 28 kilobase pairs in length and comprises of seven open reading frames (ORF), including ORF1a and b genes that constitute the 5′ two thirds of the genome and encode the replication complex; the spike (S) gene that governs viral entry; the envelop (E), membrane (M), and nucleocapsid (N) genes that are responsible for virion assembly; and the accessory ORF3 gene with an undetermined function [2].

The porcine epidemic diarrhea virus is the causative agent of porcine epidemic diarrhea (PED), a historic, highly contagious enteric swine disease characterized by diarrhea, dehydration, and the growth retardation in pigs of all ages [1]. In late 2010, new and highly virulent PEDV strains arose in China and spread rapidly worldwide by late 2013, resulting in nearly 100% mortality in the affected nursing piglets [3,4,5]. To date, there are still indelible endemics and considerable economic losses in the global swine market [6]. Besides, the protection conferred by currently available vaccines is, unfortunately, unsatisfactory [6,7]. Based on the nucleotide identity of the S gene, PEDVs are categorized into four genogroups (Gs), namely G1a, G1b, G2a, and G2b [8]. Among these, the G2b PEDV strains that predominate the field in Asia and North America, show higher pathogenicity [9] and appear to elicit broader protection across different genogroups [10,11,12]. Although the increased virulence of new PEDV strains was ascribed to several mutations in the S gene through viral escape from antibody neutralization induced by traditional vaccines [13,14], the detailed mechanism remains elusive.

Previously, we generated an attenuated G2b Taiwan PEDV strain, PEDV Pintung 52 passage 96 (PEDVPT-P96) virus, by serial passage of the parental PEDVPT strain in Vero cells [15]. Despite the high potential of PEDVPT-P96 as a future vaccine candidate against PEDV as indicated by its reduced pathogenicity and robust host immune response in our 5-week-old piglet model [15], the difficulty in PEDV isolation and subsequent lengthy passage process rendered the PEDVPT-P96 unable to promptly respond to the vast outbreak in late 2013. This year, Zhou et al. [16] reported a new disastrous swine disease outbreak in China in 2016 caused by an HKU2-related coronavirus of bat origin, again highlighting the potential burden of the interspecies jumping of coronavirus, and a pressing need for a readily applicable vaccine platform for new emergences.

The reverse genetics system has been widely used to study viral pathogenesis and novel vaccine design. At present, the reported PEDV infectious cDNA clones were exclusively constructed based on sequences of the representative wild-type PEDV isolates [17,18,19,20,21]. Consequently, they are highly pathogenic and fatal in suckling piglets, comparable to their parental viruses. With regard to vaccine use, further genetic editing is necessary to attenuate these recombinant viruses. Alternatively, a complementary approach exploiting the attenuated phenotype to address the safety concerns has not been described. In the present study, a full-length cDNA clone of the attenuated PEDVPT-P96, iPEDVPT-P96, was generated. In addition, the pathogenicity, immunogenicity, and protection against virulent PEDVPT-P5 challenge by iPEDVPT-P96 were evaluated in the 5-week-old piglet model. Our data suggest that the iPEDVPT-P96 virus was more attenuated but able to elicit similar immunogenicity and immunoprotection against the autologous virulent PEDVPT-P5 challenge compared to the parental PEDVPT-P96 virus. This iPEDVPT-P96 cDNA clone is expected to allow for the manipulation of the viral genome to study viral pathogenesis. On the other hand, it can serve as a vaccine platform, for example, by directly replacing the S gene with that of the (re)emerging swine coronaviruses.

## 2. Materials and Methods

### 2.1. Cells and Viruses

Vero C1008 cells (ATCC No. CRL-1586) were maintained in Dulbecco’s modified Eagle’s medium (DMEM, Gibco, Grand Island, NY, USA) supplemented with 10% fetal bovine protein (FBS), 250 ng/mL Amphotericin B, 100 U/mL Penicillin, and 100 μg/mL Streptomycin. Viral stock of PEDVPT-P96 in post-inoculation medium (PI medium) containing DMEM supplemented with 0.3% tryptose phosphate broth (TBP), 0.02% yeast extract (0.02%), and 10 μg/mL trypsin, as prepared in our previous study [15], was used for generating the infectious cDNA clone and as the control for in vivo and in vitro studies, whereas the virulent PEDVPT-P5 virus was used for animal challenge.

### 2.2. Generation of the Full-Length cDNA Clone, iPEDVPT-P96

**Figure 1:**
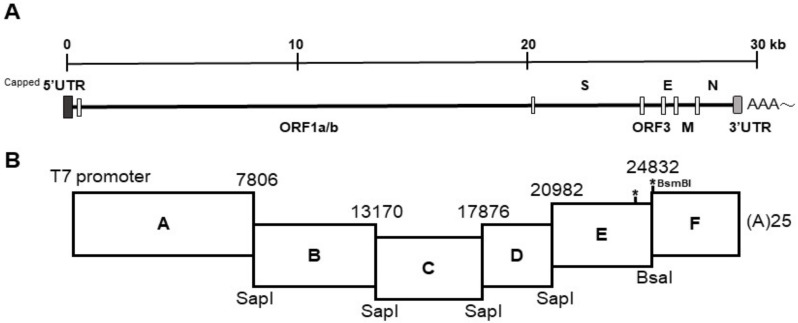
The schematic diagram of the PEDV genome and the cloning strategy for construction of the infectious PEDVPT-P96 clone. (**A**) Organization of the complete PEDV genome. (**B**) Six fragments were joined with specific restriction enzymes as labeled and the corresponding nucleotide sites are indicated. The representative sites of introduced silent mutations are marked with asterisks and the inserted *Bsm*BI site was labeled.

The strategy used to engineer the full-length cDNA clone of PEDVPT-P96, namely iPEDVPT-P96, was modified according to a previously published method [22]. Briefly, the complete genome of PEDVPT-P96 (Genbank accession No. KY929406) was divided into six fragments by PCR amplification using primer pairs (Table 1) incorporated with specific type-IIS restriction enzyme sites for seamless ligation. Fragment A contained a T7 promoter sequence at its 5′ end to allow in vitro transcription and the sequence was designed following the article published previously [19]; a 25-adenosine sequence was added to the 3′ end of fragment F to simulate polyadenylation. A naturally occurring *Bsa*I site in fragment E was removed by introducing a silent mutation (C24341T) by site-directed mutagenesis according to the previously described protocol [23]. An additional synonymous mutation (T24841C) was generated near the junction of fragments E and F, to create a novel *Bsm*BI recognition site. PCR amplicons of all fragments were subcloned into plasmid vectors (pJET1.2; Thermo Fisher Scientific, Waltham, MA, USA). Each subclone was digested with the corresponding type-IIS restriction enzymes as indicated in Figure 1 and gel-purified using a QIAquick Gel Extraction Kit (Qiagen, Hilden, Germany). Assembly of the full-length cDNA was conducted by employing T4 ligase (NEB, Ipswich, MA, USA) overnight at 4 °C. The ligated full-length cDNA was phenol-chloroform extracted and in vitro transcribed to full-length RNA transcripts using a mMessage mMachine T7 transcription kit (Ambion, Austin, CA, USA) following the manufacturer’s instructions. The cap analog to GTP ratio was adjusted to 1:1 to increase the yield of full-length transcripts. To facilitate viral recovery, nucleocapsid (N) transcripts were also generated from amplicons flanking the entire N gene with the addition of the T7 promoter sequence and poly-A tail at the 3′ and 5′ ends, respectively. The N transcripts were precipitated using lithium chloride (Ambion, Austin, CA, USA) and purified with ethanol.

### 2.3. Recovery of the Full-Length cDNA Clone of iPEDVPT-P96

The reaction mixture of full-length transcripts (30 μL) and 5 μg of N transcripts were mixed thoroughly and electroporated into Vero cells in a suspension of 800 μL with 10^7^ cells/mL in RNase-free phosphate buffered saline (PBS) using a Gene Pulser Xcell™ Electroporation System (Bio-Rad, Hercules, CA, USA), with four pulses at 450 V, 50 μF and 2–3 s rest between each pulse. After electroporation, the cells were initially incubated at room temperature for 15 min and then resuspended in DMEM supplemented with 10% FBS in a 75-cm^2^ flask overnight at 37 °C to allow for recovery. On the next day, the cells were washed twice with Dulbecco's phosphate-buffered saline (DPBS) and maintained in PI medium for an additional 2–3 days until cytopathic effects (CPE) characterized by cell fusion, syncytial cell formation, and cell detachment were observed. The whole flask was subjected to one freeze-and-thaw cycle and the rescued virus was passaged once to generate a viral stock of iPEDVPT-P96 for further use. The viral stock was titrated in Vero cells in a 96-well plate to determine viral titer.

### 2.4. In Vitro Characterization of the Full-Length cDNA Clone of iPEDVPT-P96

#### 2.4.1. Immunocytochemistry

To detect the PEDV antigen, immunocytochemistry (ICC) was performed as described previously [15]. Briefly, Vero cells infected with iPEDVPT-P96 showing typical CPE were fixed with 80% ice-cold acetone, air-dried, and incubated with an in-house anti-PEDV N antibody at room temperature (RT) for 1 h. After washing three times with PBS, a polyclonal anti-rabbit/mouse immunoglobulin, EnVision-DAB^+^ system (Dako, Carpinteria, CA, USA), was applied for 1 h at RT. Following three washes with PBS, the cells were incubated with 3, 3′-diaminobenzidine (DAB) chromogen from a peroxidase DAB substrate kit (Dako, Carpinteria, CA, USA) according to the manufacturer’s instructions. Positive signals were visualized under an inverted light microscope (Nikon, Tokyo, Japan).

#### 2.4.2. Sequence Analysis

Sequence analysis was conducted as described previously [15] and a primer pair (SF5 and N-4 listed in Table 1) targeting the genome between nucleotides 23037 to 25574 was used to identify the presence of marker mutations, C24341T and T24841C, in the iPEDVPT-P96 viral stock.

#### 2.4.3. Comparison of Growth Kinetics and Plaque Morphologies between PEDVPT-P96 and iPEDVPT-P96

The growth characteristics of iPEDVPT-P96 and PEDVPT-P96 in Vero cells were evaluated and compared by examining the growth kinetics and plaque morphologies. Confluent monolayers of Vero cells were prepared on 6-well plates and infected with each virus at the multiplicity of infection (MOI) 0.01 for 1 h at 37 °C in triplicate. To determine the growth kinetics, cells were washed twice with DPBS and then maintained in the PI medium. The supernatants at indicated time points, 0, 12, 24, and 36 h post-inoculation (HPI), were collected and subjected to titration in Vero cells seeded in 96-well plates. Plaque assays were performed to characterize the plaque morphologies. After the adsorption of PEDVs at MOI 0.01, Vero cells were washed twice with DPBS and overlaid with PI medium containing 1% agarose (Invitrogen, Carlsbad, USA) pre-warmed to 42 °C. Upon solidification of the overlays, the plates were incubated for 3 days at 37 °C to allow PEDV-infected Vero cells to produce distinct plaques. The cells were fixed in 3.16% neutral formalin for 1 h at RT. The semisolid overlays were then removed manually and the cells were stained with 1% crystal violet in 20% ethanol and distilled water for 1 min. The viral plaques were inspected after washing off the crystal violet, rinsing the plates with water, and air-drying at RT. The diameters of representative plaques for each virus were measured and compared.

### 2.5. Animal Experiment

Fifteen, 4-week-old, Large White × Duroc, crossbred piglets that were PEDV-seronegative and PEDV-shedding negative were selected from a conventional pig farm with no history of a G2b PEDV strain infection. These piglets were randomly assigned to three groups, including the PEDVPT-P96 group (*n* = 5), iPEDVPT-P96 group (*n* = 5), and mock group (*n* = 5), and acclimated for one week prior to inoculation. At 5 weeks of age, the piglets in each group were orally inoculated with 4 mL of 5 × 10^5^ TCID_50_/mL of PEDVPT-P96, 5 × 10^5^ TCID_50_/mL of the PEDVPT-P96 virus, or PI medium, respectively. To evaluate the protective efficacy induced by iPEDVPT-P96, piglets at 9 weeks of age in all groups were orally challenged with 5 mL 10^5^ TCID_50_/mL of PEDVPT-P5. The animal experimental procedure was reviewed and approved by the Institutional Animal Care and Use Committee (IACUC) of the National Taiwan University (Taiwan, Republic of China) with approval No.: NTU105EL-00160.

#### 2.5.1. Clinical Signs and Body Weight

Fecal consistency was monitored daily and scored visually as 0 = normal, 1 = loose, 2 = semi-fluid, and 3 = watery, as described previously [15]. The body weight of each piglet was measured weekly.

#### 2.5.2. Detection of Fecal PEDV Viral Load

To quantitate the viral RNA in stools, fecal samples collected from rectal swabs were resuspended in 1000 μL DPBS, pulse-vortexed for 5 s and precipitated by centrifugation at 13,000 rpm for 5 min. RNA was extracted automatically from 200 μL of stool suspension on a QIAcube using the Cador Pathogen 96 QIAcube HT Kit (Qiagen, Hilden, Germany) according to the manufacturer’s instructions. cDNA was reverse-transcribed using a QuantiNova Reverse Transcription Kit (Qiagen, Hilden, Germany) following the manufacturer’s protocols and it was used for quantitative real-time PCR analysis (qPCR). qPCR was conducted using the previously published primer-probe set [4] and a QuantiNova^®^ Probe PCR Kit (Qiagen, Hilden, Germany) on a CFX96 Thermal Cycler (Bio-Rad, Hercules, CA, USA). The thermal profile comprised an initial denaturation at 95 °C for 2 min followed by 45 cycles of 95 °C for 15 s followed by 60 °C for 15 s. The detection limit of the assay was determined by generating standard curves from serial 10-fold dilutions of known amounts of in vitro transcribed RNA followed by reverse transcription and qPCR quantification as described above. The detection limit was calculated as 100 RNA copies per mL.

#### 2.5.3. Detection of PEDV-Spike Specific Plasma IgG and Fecal and Salivary IgA

To detect PEDV specific plasma IgG and fecal and salivary IgA, an in-house, PEDV S protein based indirect enzyme-linked immunosorbent assay (ELISA) was conducted as described in the previous study [24]. In brief, 96-well, flat-bottom microtiter plates (Nunc, Roskilde, Denmark) were coated with 2 μg/mL purified recombinant PEDVPT S protein (200 ng/well) and incubated overnight at 4 °C. The plates were washed six times with 100 μL of PBST (PBS containing 0.05% Tween 20) and then blocked with 300 μL of blocking buffer (1% bovine serum albumin in PBS) at RT for 1 h. For the detection of plasma IgG, 100 μL of 40-fold diluted plasma samples in blocking buffer were added following six washes and incubated at RT for 1 h. For fecal and salivary IgA, 100 μL of eluted fecal suspension and saliva at 1:2 dilution in blocking buffer were added following six washes and kept overnight at 4 °C. After incubation, the samples were discarded and the plates were washed six times. To detect plasma IgG, and the fecal and salivary IgA, 100 μL of either horseradish peroxidase (HRP) conjugated goat anti-pig IgG (Kirkegaard & Perry Laboratories, Milford, MA, USA) at 1:1000 dilution, or goat-anti-pig IgA (Abcam, Cambridge, UK) diluted 1:10,000 were added, respectively, and incubated at RT for 1 h. Following a wash step, 50 μL of tetramethylbenzidine (TMB) substrate solution (Kirkegaard and Perry Laboratories) was added to allow color development at RT for 10 min. The reactions were terminated by adding 50 μL of TMB stop solution (Kirkegaard and Perry Laboratories) to each well. The optical density (OD) at 405 nm was measured on an ELISA reader (Molecular Devices, Sunnyvale, CA, USA). The antibody titers were expressed as sample-to-positive control ratio (S/P ratio) values.

#### 2.5.4. Viral Neutralization (VN) Assay

Plasma samples of piglets were heated at 56 °C for 30 min to inactivate complement prior to use. For each well, mixtures containing 50 μL of PEDVPT-P5 virus (50 viral particles) and 50 μL of 2-fold diluted plasma samples in PI medium were incubated at 37 °C for 1 h before applying to Vero cells (2 × 10^4^/well). After incubation with the virus-plasma mixture for 1 h, Vero cells were washed twice and maintained in PI medium for 24 h. Cytopathic effects were detected using inverted light microscopy (Nikon, Tokyo, Japan). The neutralizing titer was defined as the highest dilution without CPE.

### 2.6. Statistical Analysis

The results of body weight, antibody titers, viral titer of growth kinetics at each time point, and fecal viral shedding were analyzed statistically on GraphPad Prism 6.0 (GraphPad Software, San Diego, CA, USA) with Two-way ANOVA by time. A *p* value less than 0.05 was considered statistically significant.

## 3. Results

### 3.1. Generation and Recovery of the Full-Length cDNA Clone of iPEDVPT-P96

**Figure 2:**
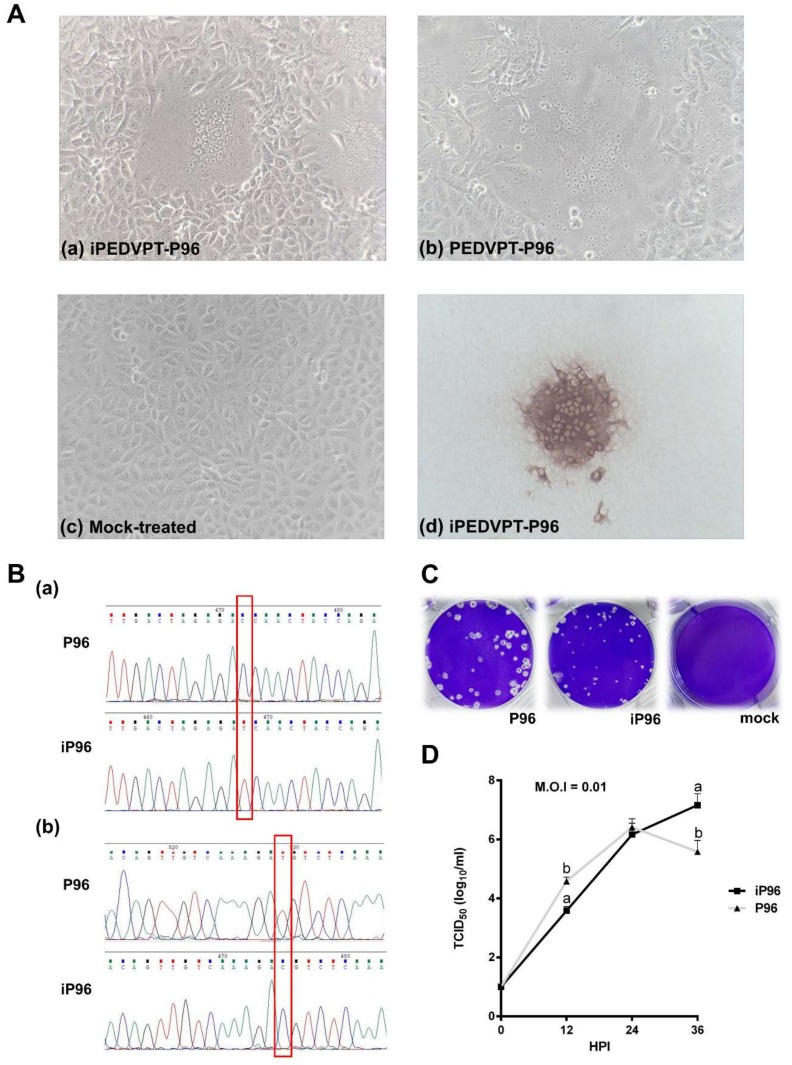
The in vitro characterization of the recombinant iPEDVPT-P96 virus. (**A**) The cytopathic effect in Vero cells infected with the iPEDVPT-P96 virus (**Aa**), PEDVPT-P96 virus (**Ab**), or mock-treated (**Ac**), and the immunocytochemistry (**Ad**) detecting PEDV N protein. The microphotographs were taken at 400× magnification. (**B**) Sequence analysis indicated the appropriate marker mutations of cysteine to thymidine at nucleotide site 24341 and, thymidine to cysteine at nucleotide site 24841, as indicated by the red boxes. (**C**) Plaque morphologies of Vero cells infected with the iPEDVPT-P96 virus, PEDVPT-P96 virus, or mock-infected, acquired after three days of incubation. Note that the iPEDVPT-P96 infection resulted in more consistent plaque sizes. (**D**) The growth kinetics of the iPEDVPT-P96 and PEDVPT-P96 viruses in the Vero cells after infection at 0.01 MOI. The titers of each virus at the indicated time points (hours post-infection; HPI) were expressed as the mean ± standard deviation. Different alphabetic letters indicated significant differences between the iPEDVPT-P96 or PEDVPT-P96 groups (*p* < 0.05).

To generate the full-length cDNA clone of iPEDVPT-P96, the complete genome sequence of PEDVPT-P96 was split into six fragments by designing primer pairs fused with specific type-IIS restriction enzymes. After the restriction enzyme digestion and DNA ligation, transcripts containing the full-length iPEDVPT-P96 sequence were successfully prepared. Typical PEDV-associated cytopathic effects in Vero cells, characterized by multinucleated giant syncytia, were observed at about one day post-electroporation (Figure 2A). The presence of iPEDVPT-P96 was confirmed by the detection of the PEDV N protein by immunocytochemistry (Figure 2A) and sequence analyses for identification of the introduced substitutions, C24341T and T24841C (Figure 2B). After an additional day, recombinant viral supernatants were harvested when the CPE reached 90% of the confluent monolayer. An iPEDVPT-P96 viral stock with a titer of 5.62 × 10^6^ TCID_50_/mL was prepared by an additional passage of the viral supernatants in the Vero cells.

### 3.2. Characterization of the iPEDVPT-P96 Virus In Vitro

To characterize the recombinant iPEDVPT-P96, plaque morphologies and growth kinetics of the recombinant iPEDVPT-P96 in Vero cells were compared to those of the parental PEDVPT-P96 virus. Interestingly, aside from the constantly short diameter, the plaque size of iPEDVPT-P96 appeared to be more uniform than that of PEDVPT-P96 (Figure 2C). As to the replicative capacity, the multistep growth kinetics of both the iPEDVPT-P96 and PEDVPT-P96 viruses in Vero cells were examined. At MOI of 0.1, while PEDVPT-P96 replicated rapidly to the titer of 4.58 ± 0.14 TCID_50_/mL at 12 HPI, reached the peak viral titer of 6.42 ± 0.29 TCID_50_/mL at 24 HPI, and showed a declined viral titer of 5.58 ± 0.38 TCID_50_/mL at 36 HPI. iPEDVPT-P96 showed a steady increase in viral progenies and a significantly decreased viral titer of 3.58 ± 0.14 TCID_50_/mL at 12 HPI, a comparable viral titer of 6.16 ± 0.38 TCID_50_/mL at 24 HPI, and a significantly high viral titer of 7.16 ± 0.38 TCID_50_/mL at 36 HPI (Figure 2D). At MOIs of 0.001 and 0.00001, similar to those observed at 0.01 MOI, iPEDVPT-P96 replicated slower in the beginning, reached a similar peak viral titer as compared to PEDVPT-P96 and showed a relatively slower reduction of viral titer after reaching the peak titer (see Figure S1).

### 3.3. Comparison of the Pathogenicity of iPEDVPT-P96 and PEDVPT-P96 Viruses

**Figure 3:**
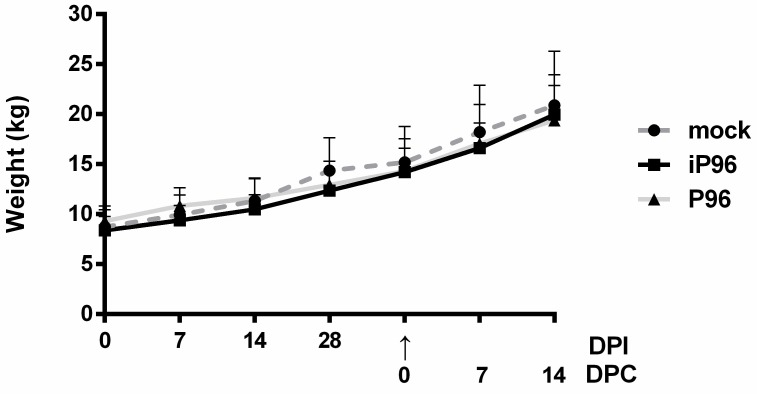
The weekly change in the mean body weight of 5-week-old piglets inoculated with the iPEDVPT-P96 virus (■), the PEDVPT-P96 virus (▲), or mock-inoculated (●) through the study. Each group consists of 5 piglets. The error bars indicated the standard deviation. The arrow indicated the time point (28 DPI or 0 DPC) of challenge with the PEDVPT-P5 virus. iP96: PEDVPT-P96; PEDVPT-P5; P96: PEDVPT-P96 virus; mock: PI medium control; DPI: days post-inoculation; DPC: days post-challenge.

**Figure 4:**
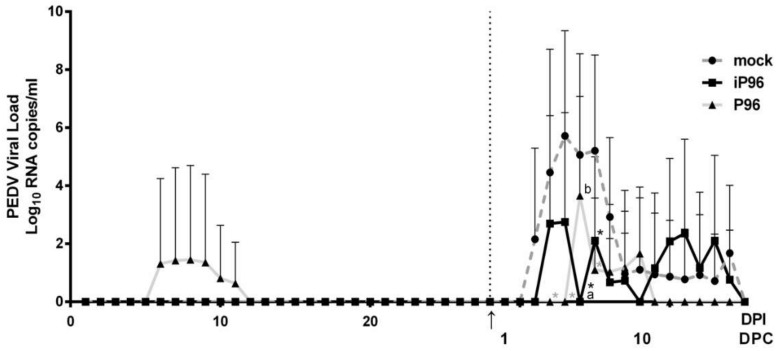
The mean value of RNA copies in the feces of piglets inoculated with the iPEDVPT-P96 virus (■), the PEDVPT-P96 virus (▲), or mock-inoculated (●) through the study. Each group consists of 5 piglets. The dashed line and arrow indicated the time point (28 DPI or 0 DPC) at which pigs were challenged with the PEDVPT-P5 virus. The error bars indicated standard deviation. Data were analyzed using two-way ANOVA by time. Significant differences with the mock group were labeled with asterisks (*p* < 0.05). Different alphabetic letters indicate significant differences between the iPEDVPT-P96 or PEDVPT-P96 groups (*p* < 0.05). iP96: PEDVPT-P96; PEDVPT-P5; P96: PEDVPT-P96 virus; mock: PI medium control; DPI: days post-inoculation; DPC: days post-challenge.

To evaluate the pathogenicity of the iPEDVPT-P96 virus, 5-week-old crossbred piglets were orally inoculated with 4 mL of 5 × 10^5^ TCID_50_/mL of the iPEDVPT-P96 virus, PEDVPT-P96 virus, or PI medium. The clinical impact of viral inoculation in each piglet was evaluated by daily clinical fecal scoring, fecal viral shedding, and weekly body weight changes. During the study, no significant difference in body weight was revealed among the different groups at any indicated time points (Figure 3). While one piglet in the PEDVPT-P96 group showed intermittent loose diarrhea (score = 1) and viral shedding at 6 to 11 days post-inoculation (DPI) with the peak viral titer of 1.45 ± 3.24 log_10_ RNA copies/mL at 8 DPI (Figure 4), no evidence of PEDV-associated clinical signs and fecal viral shedding were demonstrated in both iPEDVPT-P96 and mock groups.

### 3.4. PEDV-Specific Systemic and Mucosal Antibody Responses Induced by iPEDVPT-P96 Inoculation

**Figure 5:**
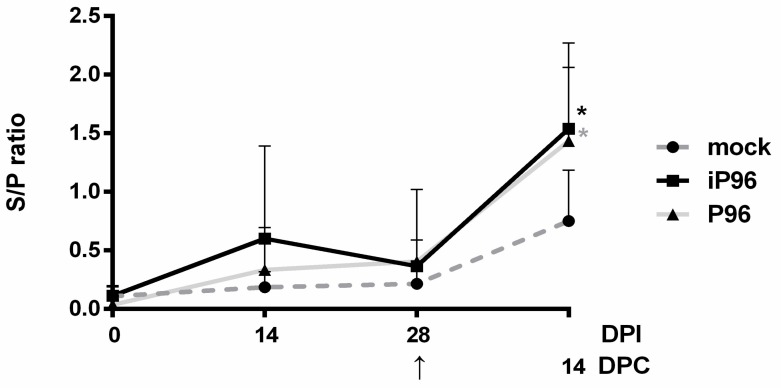
The mean sample-to-positive ratio of the plasma PEDV spike (S)-specific IgG in piglets inoculated with the iPEDVPT-P96 virus (■), the PEDVPT-P96 virus (▲), or mock-inoculated (●) at 0, 14, and 28 days post-inoculation (DPI) and at 14 days post-challenge (DPC) after re-challenge or challenge with PEDVPT-P5 virus. Each group consisted of 5 piglets. Data are expressed as the mean ± standard deviation of the sample-to-positive ratio (S/P ratio) in each group generated from optical density (OD) values obtained by an in-house PEDV-Spike based indirect ELISA. The arrow indicated the time point (28 DPI or 0 DPC) of challenge with PEDVPT-P5 virus. Statistically significant differences between each group were examined using two-way ANOVA by time. Significant differences from the mock group are labeled with asterisks (*p* < 0.05).

**Figure 6:**
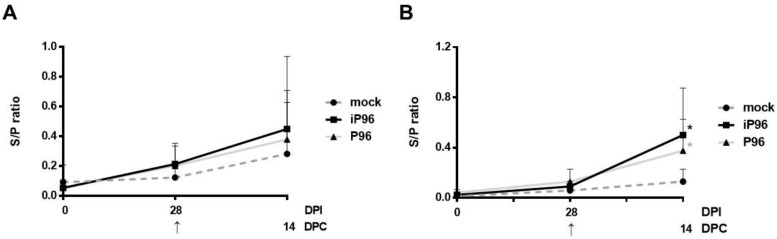
The mucosal immunity of piglets induced by the iPEDVPT-P96 virus (■), the PEDVPT-P96 virus (▲), or mock-inoculated (●) at 0 and 28 days post-inoculation (DPI) and at 14 days post-challenge (DPC), after re-challenge or challenge with the PEDVPT-P5 virus. Each group consisted of 5 piglets. The mean S/P ratios of fecal (**A**), and salivary (**B**) anti-PEDV spike IgA in each group at a given time point were generated from the original optical density (OD) values using an in-house PEDV-Spike based indirect ELISA. The error bars indicate standard deviation. The arrow indicated the time point (28 DPI or 0 DPC) of challenge with PEDVPT-P5 virus. Statistically significant differences between each group were examined using a two-way ANOVA by time. Significant differences from the mock group are labeled with asterisks (*p* < 0.05).

After the oral inoculation of piglets with the iPEDVPT-P96 and PEDVPT-P96 viruses, systemic PEDV-specific IgG in blood and mucosal specific IgA in feces and saliva were measured at the indicated time points. Compared to the mock group, an elevated mean S/P ratio of blood PEDV-specific IgG was detected at 14 DPI and sustained until 28 DPI in piglets from both the iPEDVPT-P96 and PEDVPT-P96 groups (Figure 5). Moreover, subtle increases in the mean S/P ratios of fecal and salivary PEDV-specific IgA were also observed in both the iPEDVPT-P96 and PEDVPT-P96 groups compared to those of the mock group at 28 DPI (Figure 6).

### 3.5. Immunoprotection against the Virulent PEDVPT-P5 by iPEDVPT-P96

**Figure 7:**
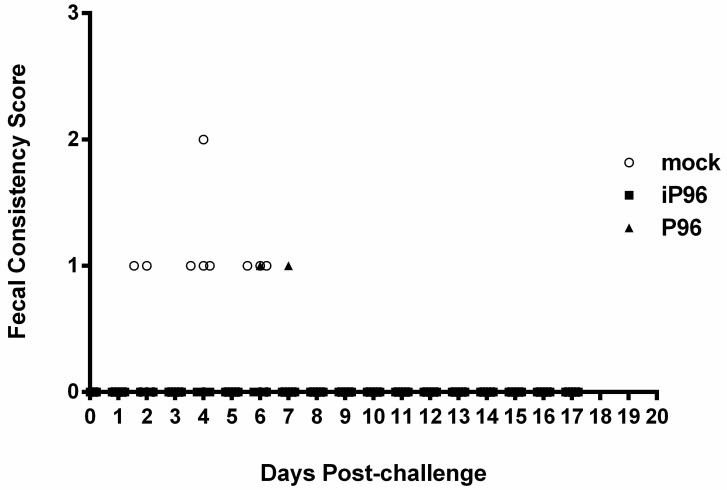
The fecal consistency score of piglets inoculated with the iPEDVPT-P96 virus (■), the PEDVPT-P96 virus (▲), or the mock-inoculated (○) after challenge with PEDVPT-P5. Each group consists of 5 piglets. The fecal consistency sign of each piglet was scored visually as 0 = normal, 1 = loose, 2 = semi-fluid, and 3 = watery.

After oral inoculation with the virulent PEDVPT-P5 virus, the daily fecal viral sheddings and fecal consistencies in all groups were evaluated. Similar to our previous data [15], all piglets in the mock group showed the differing severity of clinical signs for diarrhea at 3-6 days post-challenge (DPC) (Figure 7). The mean value of the PEDV RNA copies in feces of the mock group became detectable at 3 DPC, shot up to a peak titer of 5.72 ± 3.62 log_10_ RNA copies/mL at 5 DPC, and then declined to a constantly low viral load of approximately 1 log_10_ RNA copies/mL at 9–16 DPC. In the PEDVPT-P96 group, two piglets showed loose diarrhea (score = 1) for one day at 6 and 7 DPC, respectively. The onset of fecal viral shedding in the PEDVPT-P96 group after the challenge was further delayed to 6 DPC compared to the other two groups. The highest viral titer of the PEDVPT-P96 group also appeared at 6 DPC, calculated as 3.66 ± 3.41 log_10_ RNA copies/mL. In the iPEDVPT-P96 group, all pigs remained clinically normal without an observation of diarrhea. Compared to the mock group, only three of the five pigs in the iPEDVPT-P96 group established fecal viral shedding through the experiment, with a delayed onset and a steadily fluctuated low viral shedding, ranging from the highest titer of 2.75 ± 3.77 to log_10_ RNA copies/mL to undetectable levels (Figure 5).

### 3.6. Systemic and Mucosal PEDV-Specific Antibody Responses after the PEDVPT-P5 Challenge

In the mock group, the mean value of the S/P ratio of the systemic PEDV-specific IgG titers in the blood of piglets remained unchanged prior to the PEDVPT-P5 challenge, after which the mean S/P value rose to 0.75 ± 0.43 at 14 DPC (Figure 5). On the other hand, the mean values of systemic PEDV-specific IgG in blood from the iPEDVPT-P96 and PEDVPT-P96 groups increased sharply to 1.54 ± 0.74 and 1.43 ± 0.62 at 14 DPC, respectively, which was significantly higher than that of the mock group (Figure 6). As for mucosal immunity, similar to the trend noticed in the systemic PEDV-specific IgG in blood, significant increases in the mean S/P values of both fecal and salivary anti-PEDV specific IgA were noted in the iPEDVPT-P96 and PEDVPT-P96 groups after challenge (Figure 6). Among them, the S/P values of salivary anti-PEDV specific IgA, 0.5 ± 0.33 and 0.37 ± 0.22, in the iPEDVPT-P96 and PEDVPT-P96 groups, respectively, were significantly higher than those of the mock group (0.13 ± 0.08) at 14 DPC (Figure 7).

### 3.7. Virus Neutralization Test

**Figure 8:**
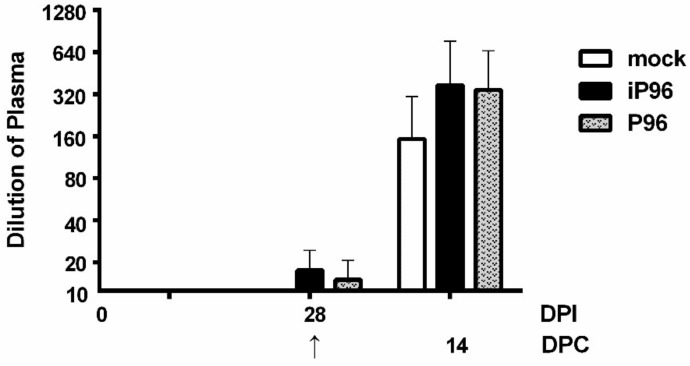
The plasma neutralizing antibody titers in piglets inoculated with the iPEDVPT-P96 virus (black), the PEDVPT-P96 virus (gray), or mock-inoculated (white) at 0 and 28 days post-inoculation (DPI) and at 14 days post-challenge (DPC) after re-challenge or challenge with the PEDVPT-P5 virus. Each group consisted of 5 piglets. The arrow indicated the time point (28 DPI or 0 DPC) of challenge with PEDVPT-P5 virus. Data were expressed as the mean ± standard deviation. Statistically significant differences between each group were examined using a two-way ANOVA by time.

The VN antibody titers of piglets in each group are depicted in Figure 7. Similar to the trend observed in systemic plasma IgG titers, the iPEDVPT-P96 group showed a comparable VN antibody titer against PEDVPT-P5 to that of the PEDVPT-P96 group through the study. The mean VN antibody titers of both groups increased mildly at 28 DPC and spectacularly at 14 DPC and were higher than those of the mock group during the study (Figure 8).

## 4. Discussion

In the present study, we described the first development of an infectious cDNA clone of iPEDVPT-P96, and evaluated it’s in vitro and in vivo characteristics. Compared to the parental PEDVPT-P96 virus, iPEDVPT-P96 replicated more slowly in the beginning and reached a similar peak viral titer with similar but more uniform plaque sizes, suggesting that the composition of viral quasispecies in iPEDVPT-P96 is less complex than that in the original PEDVPT-P96 stock. Moreover, neither fecal PEDV RNA shedding nor a PEDV-associated clinical illness was detected in conventional 5-week-old piglets inoculated with the iPEDVPT-P96 virus, indicating a further attenuated phenotype in vivo. Importantly, piglets in the iPEDVPT-P96-inoculated group showed comparable levels of anti-PEDV specific plasma IgG, fecal/salivary IgA, plasma neutralizing antibody titers, and a weakened but modest immunoprotection against the virulent PEDVPT-P5 challenge compared to the parental PEDVPT-P96-inoculated piglets. Taken together, our results suggest that iPEDVPT-P96 is immunogenic in piglets and could be a potential safe viral vector candidate for vaccine development.

While inoculation with iPEDVPT-P96 was demonstrated to completely protect the piglets from developing diarrhea after challenge with the virulent PEDVPT-P5, the iPEDVPT-P96-inoculated group showed an earlier onset and longer duration of fecal PEDV RNA shedding with a higher peak value than that of the parental PEDVPT-P96-inoculated group. These data suggested that iPEDVPT-P96 conferred a relatively weakened protection than that of the parental PEDVPT-P96. Considering that the major variation between the PEDVPT-P96 and iPEDVPT-P96 viruses should be the heterogeneity of viral population as noted in plaque assays wherein the iPEDVPT-P96 virus produced more uniform plaques than those of the parental PEDVPT-P96 virus in Vero cells, we speculate that the decrease in quasispecies diversity in iPEDVPT-P96 may partly contribute to its further attenuation in vivo. To investigate the speculation, we conducted the next generation sequencing (NGS) to determine the quasispecies diversity of both viruses by exploring the recovered variants against our previous published sequence of PEDVPT-P96 generated by Sanger sequence (see Table S1). The results clearly demonstrated that the PEDVPT-P96 carried a greater sequence diversity than that of the iPEDVPT-P96, including 23 single nucleotide variants (SNV) and resultant 16 amino acid substitutions; of interest, seven SNV were found in the spike gene and 3 of them were the same as the virulent PEDVPT-P5. For iPEDVPT-P96, excluding the artificially introduced marker mutations and those derived from the SNV of PEDVPT-P96 upon the initial construction of subclones of iPEDVPT-P96, only 3 SNV were uncovered. Indeed, generating viruses with high-fidelity replication has been proposed as a rational strategy to develop genetically stable and safe attenuated vaccines [25,26]. However, for attenuated viruses, the in vivo fitness and antigenicity are already abated after serial cell culture passage. On the basis of quasispecies theory that the cooperative interplay between different variants determines viral characteristics including virulence [27,28], it is possible that the consensus sequence used for constructing iPEDVPT-P96 no longer sustained the original affinity of virus-host interaction or viral replication in enterocytes, which therefore impaired viral entry and/or limited the viral infection. Furthermore, the limited diversity of the S gene in the iPEDVPT-P96 viral stock might also diminish the potency and protective broadness of the induced antibody responses explaining the comparable level of systemic and mucosal antibody response but weakened protection in the iPEDVPT-P96-inoculated group. This speculation could be tested by comparing the tissue tropism and the quantity of PEDV antigens of both PEDVPT-P96 and iPEDVPT-P96 in enterocytes using a 7-day-old piglet model. Other explanation for the attenuation of iPEDVPT-P96 might be attributed to the possible addition of the five nucleotides “GGAGA” at the very extreme of the 5′UTR based on our primer design (see Table 1). Considering the location that these additional nucleotides sequence should neither alter the secondary structures of the SL1 stem-loop, that serve as *cis*-acting elements required for driving subgenomic RNA synthesis nor change any consensus transcription regulatory sequences 5′-XUA(A/G)AC-3′, we assumed that the effect of these additional nucleotides in the rescued iPEDVPT-P96 virus on replication might be minimal. Nonetheless, further studies are required to determine the potential effect of these five additional nucleotides on iPEDVPT-P96 replication.

In the present study, the profile of fecal PEDV RNA shedding and the pattern of antibody responses after inoculation with PEDVPT-P96 appeared milder than those observed in our previous findings despite the fact that the same viral stock was used and that the conventional piglets used in this study were purchased from the same pig farm and housed in the same animal facility as described previously [15]. That is, it seemed that the conventional piglets used in the current study were more resistant to the PEDVPT-P96 infection. Several host factors such as genetic variation, type of feed, gut microflora, and immune status among different litters at the time of inoculation may play an important role in the varying severity of clinical outcomes and immune responses [9]. Ideally, the discrepancy between the previous experiment might be minimized by expanding the size of groups, particularly if we chose conventional piglets as our target animals. Nevertheless, due to the difficulty in collecting large numbers of PEDV-negative piglets in Taiwan where PED has become endemic, we decided to use five piglets per treatment to reach a statistical effect practice. Although the use of conventional pigs often magnifies those variables and results in higher variation in the experimental results, to mimic the pathogenicity and immunoprotection of PEDV in field conditions, the conventional pig model is still a preferred as a preclinical trial model.

Since the sudden appearance of the severe acute respiratory syndrome-coronavirus (SARS-CoV) in the early 21st century, the emergence and re-emergence of coronaviruses continually threatens the global public and animal health by causing severe illnesses, by having a high potential of zoonosis, and by causing great economic losses, indicating a desperate need for an effective and readily responsive vaccine platform. The large genome size of coronaviruses warrants a great tolerance for foreign genes and subsequent expression of heterologous antigens [29,30]. In addition, the insertion of other antigens by replacing the accessory ORF3 gene or creating a novel expression cassette is also an attractive approach to design multivalent vaccines [17,18,21]. Under this concept, iPEDVPT-P96 could be a potential safe vaccine backbone that facilitates the prompt generation of chimeric vaccines with the induction of mucosal immunity. For instance, viral antigenicity can be manipulated by replacing the iPEDVPT-P96 S gene with that of other PEDV or emerging swine coronaviruses although the potential loss of attenuation due to the S substitution requires further clarification. However, it is noteworthy that the inherent genetic instability of coronaviruses due to high recombination and mutation frequency is also a matter of concern as it raises the possibility of virulence reversion and acquisition of new tropism [31]. Besides, the intrinsic characteristics of the gene of interest, the targeted locus within the coronavirus genome, may also affect the expression level and stability of the recombinant viruses [30]. Accordingly, further characterization of the virulence variation and genetic stability of iPEDVPT-P96 after different genetic modifications needs to be conducted.

In this article, we described the first successful construction of an attenuated G2b PEDV infectious cDNA clone of the iPEDVPT-P96 virus and demonstrated the maintenance of fitness in vitro along with further attenuation in vivo. We also proposed that the initial low quasispecies diversity and the additional 5 nucleotides at the 5′ end of iPEDVPT-P96 may contribute to a further attenuated phenotype and potentially less effective immunoprotection against the virulent strain challenge. Based on the results of antibody response, a prime-boost strategy or further optimization of iPEDVPT-P96 antigenicity is essential to induce sufficient protective immunity in all recipients. Together, the full-length cDNA clone of iPEDVPT-P96 generated herein is expected to provide an access to study the attenuation determinants of PEDVPT-P96 and establish a PEDVPT-P96-based recombinant vector as a vaccine platform for developing multivalent vaccines for PEDV and other porcine pathogens.

## Figures and Tables

**Table 1:** The primers for amplifying the PEDV fragments, N gene, site-directed mutagenesis, and sequence analysis.

Primer	Sequence (5′–3′)	Target
Primers used to generate continuous subclones of the complete PEDV genome		
5′F	**TAATACGACTCACTATAGGGAGA**ACTTAAAGAGATTTTCTATCTACGG	A
5′R	AAAAGCTCTTCATGGTAGTATGCATACGACAATC	
3F	AAAAGCTCTTCACCATACCTACTGTTTGCATTGC	B
3R	AAAAGCTCTTCACCTTGTTCAACCATTGCATC	
4F	AAAA*GCTCTTC*AAGGTATAGTTGGTGTTGTCACATTAG	C
4R	AAAA*GCTCTTC*AATGGGTTATACAGATAATCACAACC	
5F	AAAA*GCTCTTC*ACATACTGTATTGATATACAGCAGTGG	D
5R	AAAA*GCTCTTC*AAGTGTTACCGTTAGTAGCCTTATG	
SF	AAAA*GCTCTTC*AACTAATGCTACTGCGCGACT	E
SR	AAAA*GGTCTC*AGACAACTGTGTCAATCGTGTATTG	
3′F	AAAA*GGTCTC*ATGTCAAAGACGTCTCAAAGTCTG	F
3′R	*AAAAGGTCTC*ATTTTTTTTTTTTTTTTTTTTTTTTTTTGCGTATCCATATCAACACC	
Primers used for site-directed mutagenesis		
SmutF24341	GACTAGAGATCAACTACCAGATGTAATCCCAGATTACATCG	C24341T
SmutR24341	CTGGTAGTTGATCTCTAGTCAAATTGACATAGGTGACCAC	
Primer used to generate N gene transcript		
N-T7	CCATCGTAATACGACTCACTATAGATGGCTTCTGTCAGTTTTCAGG	N gene
N-tail	TTTTTTTTTTTTTTTTTTTTTTTAATTTCCTGTGTCGAAGATCTC	
Primers used for sequence analysis		
SF5	ACGCCTGTTAGTGTTGATTG	
N-4	GTGACAAGTGAAGCACAGAT	
SF7	ACTCTCGACTGGACATTC	

The T7 promoter sequence is in bold face. The type IIS restriction enzyme recognition sequences are italicized. The complementary sequence revealed upon restriction enzyme digestion are underlined.

## Supplementary Materials

Supplementary materials can be found at http://www.mdpi.com/1999-4915/10/10/543/s1.
