# Supplementary material for: The Characterization of Immunoprotection Induced by a cDNA Clone Derived from the Attenuated Taiwan Porcine Epidemic Diarrhea Virus Pintung 52 Strain
Source: Viruses. 2018 Oct 4;10(10):543. doi: 10.3390/v10100543 (PMC6213177; doi:10.3390/v10100543)
Supplement: Supplementary file 1 [file viruses-10-00543-s001.pdf]

Supplementary material and method

Bioinformatic analysis

Raw reads with good quality were aligned against host genome (UCSC chlSab2) using BWA-MEM v.0.7.17 with default settings, followed by non-host reads extraction. De novo assembly of those short non-host reads was accomplished using A5-miseq version 20160825 genome assembler software that produced several contigs. Longest contig with highest similarity against reference sequence from NCBI (GenBank: KY929406.1) were chosen for downstream analysis. The sequence similarity between reference sequence and assembled contig was calculated by the Needleman-Wunsch algorithm provided by EMBL-EBI website (https://www.ebi.ac.uk/Tools/psa/emboss_needle/nucleotide.html). Realignment of non-host reads were performed with BWA-MEM v.0.7.17 to virus reference sequence from NCBI (GenBank: KY929406.1). Reads subtraction and file conversion were carried out by the SAMtools v.1.9 and BEDTools v.2.27.1 software. Variants (SNPs/Indels) were determined by the VarScan v.2.4.3 software with the parameter setting: --min- coverage 20 --min-var-freq 0.2 --min-reads2 4 --min-avg-qual 20 - -p-value 0.05 for individual mode and --min-var-freq 0.2 --min- reads2 10 --min-avg-qual 20 --p-value 0.05 for paired mode.

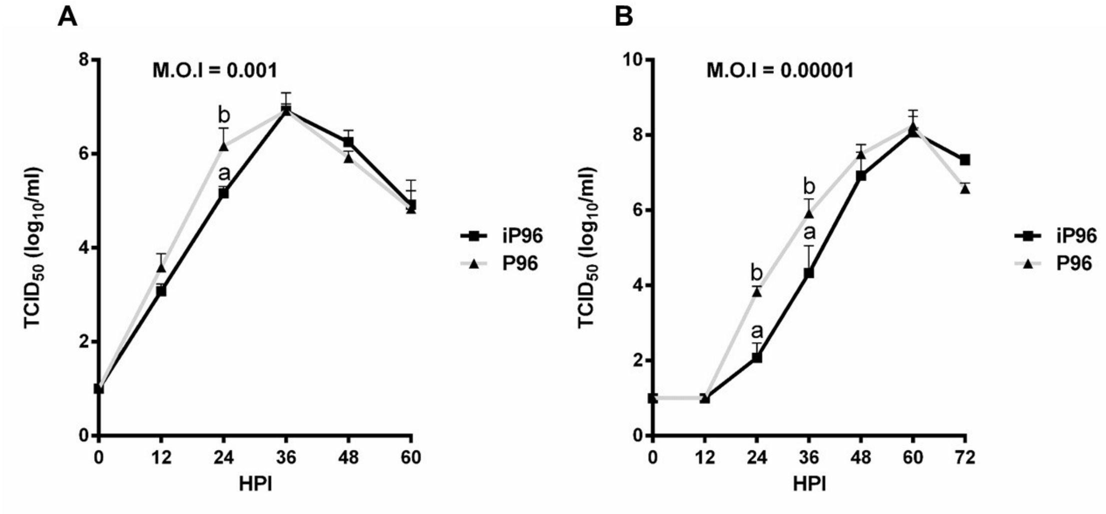

Figure S1. **Growth kinetics of iPEDVPT-P96 and PEDVPT-P96 viruses in Vero cells after infection at 0.001 and 0.00001 MOI.** The titers of each virus at the indicated time points (hours post-infection; HPI) were expressed as mean ± standard deviation. Different alphabetic letters indicate significant differences between the iPEDVPT-P96 or PEDVPT-P96 groups ($p < 0.05$).

Table S1. Variants of iPEDVPT-P96 and PEDVPT-P96 recovered from next generation sequencing

Position	Gene	Reference Base	Variant Base	Reference Amino Acid	Variant Amino Acid	Coverage Depth	Variant Reads	Variant frequency*
**PEDVPT-P96**								
46	5′UTR	A	G	--	--	4290	892	20.81%
644	NSP2	T	Y	Y	Y / H	2122	1669	78.4 %
1099	NSP2	T	G / K	N	N / K	2805	731	26.07%
1281	NSP2	C	T / Y	S	S / F	2450	684	27.93%
2151	NSP2	T	C	I	I / N	1770	1716	98.06%
3172	NSP3	C	T	D	D	3808	3188	84.38%
4983	NSP3	C	T	S	F	877	877	100%
10384	NSP6	G	T / K	Q	Q / H	3497	1322	37.81%
11950	NSP9	T	C / Y	Y	Y	4207	2737	65.06%
13597	NSP12	G	T / K	V	V / L	6133	3117	50.95%
14931	NSP12	T	G / K	V	V	4383	1629	37.17%
17064	NSP14	A	T / W	V	V	2691	935	34.78%
19470	NSP15	T	G / K	I	I / M	3160	1651	59.15%
21029	S	A	T / W	K	K / N	4191	1556	37.13%
21064	S	T	C / Y	I	I / T	3644	1391	38.17%
21931	S	A	G / R	D	D/ G	3265	1268	38.85%
22288	S	C	T / Y	S	S / L	5697	1150	20.19%
22540	S	A	R	E	E / G	6895	5727	83.06 %
23535	S	G	T / K	A	A / S	3134	1049	33.49%
23695	S	G	T	S	I	7506	5949	79.68%
24767	S	A	T / W	E	E / D	3009	605	20.11%
25517	E	T	C / Y	F	F	3321	2542	76.77%
27903	3′UTR	C	A / M	--	--	4019	912	22.71%
**iPEDVPT-P96**								
1099	NSP2	T	G[a]	N	K	1823	1823	100%
2151	NSP2	T	C[a]	I	N	917	906	100%
3172	NSP3	C	T[a]	D	D	1796	1781	99.94%
7019	NSP3	T	C	C	R	1675	1652	99.82%
16305	NSP13	T	G / K	S	S	1140	545	47.85%
24341	S	C	T[§]	D	D	3197	3173	99.94%
24767	S	A	T[a]	E	D	1367	1366	100%
24841	ORF3	T	C[§]	F	F	1061	1037	100%
25517	E	T	C[a]	F	F	1943	1912	100%
27874	3′UTR	A	G	--	--	4057	3980	99.92%

[*] The frequency was referred to the first nucleotide before the slash in column "Variant Base"
[§] Introduced marker mutation
[a] Single nucleotide variant also found in PEDVPT-P96
